# Silver Nanoparticles Stabilised by Cationic Gemini Surfactants with Variable Spacer Length

**DOI:** 10.3390/molecules22101794

**Published:** 2017-10-23

**Authors:** Martin Pisárčik, Josef Jampílek, Miloš Lukáč, Renáta Horáková, Ferdinand Devínsky, Marián Bukovský, Michal Kalina, Jakub Tkacz, Tomáš Opravil

**Affiliations:** 1 Department of Chemical Theory of Drugs, Faculty of Pharmacy, Comenius University, Kalinčiakova 8, Bratislava SK-83232, Slovakia; lukac@fpharm.uniba.sk (M.L.); horakova@fpharm.uniba.sk (R.H.); devinsky@fpharm.uniba.sk (F.D.); 2 Department of Pharmaceutical Chemistry, Faculty of Pharmacy, Comenius University, Bratislava SK-83232, Slovakia; josef.jampilek@gmail.com; 3 Faculty of Pharmacy, Comenius University, Kalinčiakova 8, Bratislava SK-83232, Slovakia; 4 Department of Cell and Molecular Biology of Drugs, Faculty of Pharmacy, Comenius University, Bratislava SK-83232, Slovakia; bukovsky@fpharm.uniba.sk; 5 Materials Research Centre, Faculty of Chemistry, Brno University of Technology, CZ-61200 Brno, Czech Republic; kalina-m@fch.vut.cz (M.K.); tkacz@fch.vut.cz (J.T.); opravil@fch.vutbr.cz (T.O.)

**Keywords:** gemini surfactant, surfactant spacer, silver nanoparticle, nanoparticle stability, microbicidal activity

## Abstract

The present study is focused on the synthesis and investigation of the physicochemical and biological properties of silver nanoparticles stabilized with a series of cationic gemini surfactants having a polymethylene spacer of variable length. UV-VIS spectroscopy, dynamic light scattering, scanning electron microscopy and zeta potential measurements were applied to provide physicochemical characterization of the silver nanoparticles. The mean size values of the nanoparticles were found to be in the 50 to 115 nm range. From the nanoparticle size distributions and scanning electron microscopy images it results that a population of small nanoparticles with the size of several nanometers was confirmed if the nanoparticles were stabilized with gemini molecules with either a short methylene spacer (two or four −CH_2_− groups) or a long spacer (12 −CH_2_− groups). The average zeta potential value for silver nanoparticles stabilized with gemini molecules is roughly independent of gemini surfactant spacer length and is approx. +58 mV. An interaction model between silver nanoparticles and gemini molecules which reflects the gained experimental data, is suggested. Microbicidal activity determinations revealed that the silver nanoparticles stabilized with gemini surfactants are more efficient against Gram-negative bacteria and yeasts, which has a direct relation to the interaction mechanism of nanoparticles with the bacterial cell membrane and its structural composition.

## 1. Introduction

Silver nanoparticles continue to attract scientific interest due to their unique application potential in various technology areas such as photography [1], electronics [2], chemical catalysis, catalytic degradation [3,4,5,6], and others. Among the wide range of applications of silver nanoparticles, their antimicrobial and bactericidal effect are of particular importance [7,8,9,10]. They show broad-spectrum antibacterial, antiviral and fungicidal effects, even against multi-resistant pathogens [10,11,12,13,14,15,16,17]. In addition to their outstanding antimicrobial and antiflogistic properties, they have a good biocompatibility, while keeping the biotoxicity at a minimum level [15,18]. Nanosilver displays potent and antimicrobial effects with less toxic side effects than the traditional silver salt sulfadiazine [19]. Silver nanoparticles are also antimicrobially active when a part of films or biofilms [20,21]. Chemical methods of preparation of silver nanoparticles are mainly based on the reduction of silver from silver salts using various reducing agents such as sodium borohydride [22,23,24,25], trisodium citrate [26], sugars [27,28], ascorbic acid [29], or tyrosine [30]. Several studies report the preparation of silver nanoparticles from silver complexes and ionic liquids [31,32,33]. The unique features of silver nanoparticles result from their specific size, shape and stability in aqueous environments which are dependent on numerous factors such as the selected method and conditions of synthesis, the concentrations of silver ions and reducing agent, the presence of stabilizing agents, temperature, etc. The main issue related to the preparation of silver nanocolloids is their spontaneous agglomeration into oligomeric aggregates. This agglomeration tendency increases as the nanoparticle size decreases. The obvious solution to stabilize nanoparticles and thus prevent their agglomeration, is the use of stabilizers. The selection of stabilizers for silver nanoparticles covers a wide range of various types of simple and complex molecules such as sugars [28], starch [8], oligomeric calixarenes [34], cyclodextrins [35] or even biological systems [5,36,37,38,39]. A significant number of silver nanoparticle stabilizers are related to the application of polymers such as polystyrene [40], polyvinylpyrrolidone [17,22,23,24], polyisopropylacrylamide [41], hydroxyethylcellulose, polyvinylalcohol [23], and others.

Cationic surfactants represent an efficient group of silver nanoparticle stabilizers due to the ability of cationic surfactant molecules to bind to the nanoparticle surface via chemisorption, resulting in a significant stabilization effect on silver nanoparticles. Bis-cationic surfactant dimers, gemini surfactants, are of special interest for the stabilization of nanoparticles. Cationic gemini molecules are composed of two hydrophobic tails and two hydrophilic heads which are interconnected by a spacer. Compared to their single-tailed analogues, cationic gemini surfactants show lower critical micelle concentration, better adsorb at the interfaces and form a variety of specific aggregate structures with the number of aggregating molecules depending of gemini molecular structure [42,43,44]. Recently, several studies have been published which report the preparation and physicochemical properties of silver nanoparticles stabilized by single-chain surfactants of different type [45] and gemini surfactants. Silver nanoparticles capped with a cationic gemini surfactant *N,N′*-bis(dodecane-1-yl)-*N,N,N′,N′*-ethane-1,2-diyl-diaminium dibromide 12-2-12 showed smaller nanoparticle sizes around 11 nm and narrower size distributions than those stabilized with the single-chain surfactant dodecytrimethylaminium bromide [46]. Variations in the spacer molecular structure of a cationic gemini molecule were utilized to modify the stabilizing effect of gemini surfactants on silver nanoparticles. Gemini surfactant molecules with hexadecyl tails and a ethane-1,2-diyl spacer group (two −CH_2_− groups) were used for the preparation of silver and gold nanoparticles [47]. A successful stabilization of silver nanoparticles was achieved by the application of gemini surfactants with octadecyl tails and a hydroxy-based spacer *N,N′*-bis(octadecane-1-yl)-*N,N,N′,N′*-2-hydroxy-propane-1,3-diyl-diaminium dibromide 18-3OH-18, which resulted in the formation of small nanoparticles with the mean diameter around 7 nm and a narrow size distribution [48]. Small gold nanoparticles of the size between 1.3 and 10.2 nm were formed with the stabilization effect of cationic gemini surfactants with propylene spacer and a variable alkyl chain length of 12, 14, 16, and 18 carbon atoms [49]. Bis-imidazolium gemini surfactant molecules with hexadecyl chains and imidazolium heads interconnected with polymethylene spacers of the 2, 5, 6, and 12 methylene groups in length, were used for the stabilization of silver nanoparticles. Their application resulted in the formation of small nanoparticles with sizes between 3 and 6 nm [50].

In the present study, a series of cationic bis-aminium gemini surfactants with polymethylene spacers of length rangings from two to 12 methylene groups is used for the stabilization of silver nanoparticles. Along with the synthesis procedure, the physicochemical properties of the nanoparticles stabilized with gemini surfactants determined utilizing UV spectroscopy, dynamic light scattering, zeta potential measurements and scanning electron microscopy are reported. Regarding their biological properties, the microbicidal activity of silver nanoparticles stabilized with gemini molecules against Gram-positive, Gram-negative pathogens and yeasts was determined and compared with that of aqueous solutions of cationic gemini surfactants without the presence of silver nanoparticles. The acquired data has allowed us to analyse and propose the relationship between the molecular structure gemini surfactant on the one hand and the size, surface charge and the gemini outer nanoparticle layer of silver nanoparticles on the other.

## 2. Results and Discussion

### 2.1. Spectral Analysis of Gemini Surfactant-Stabilized Silver Nanoparticles

**Figure 1:**
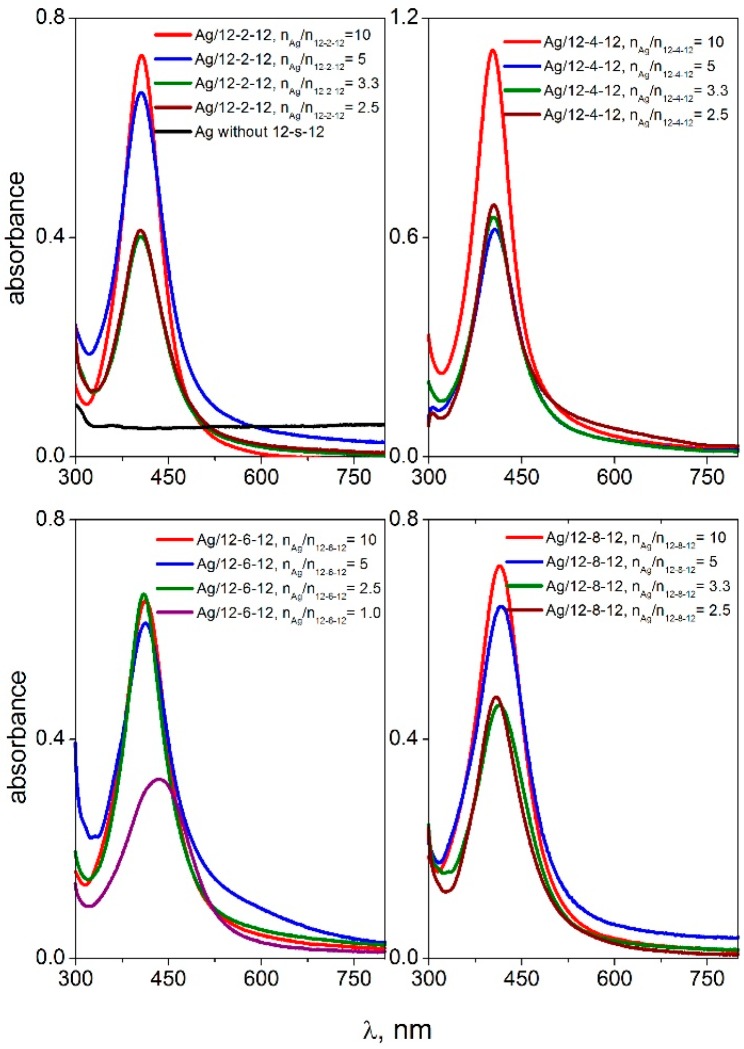
UV-VIS spectra of silver nanoparticles stabilized with gemini surfactants 12-*s*-12 with the spacer length *s* = 2, 4, 6, 8 methylene groups, at different molar ratio values n_Ag_/n_12-*s*-12_.

**Figure 2:**
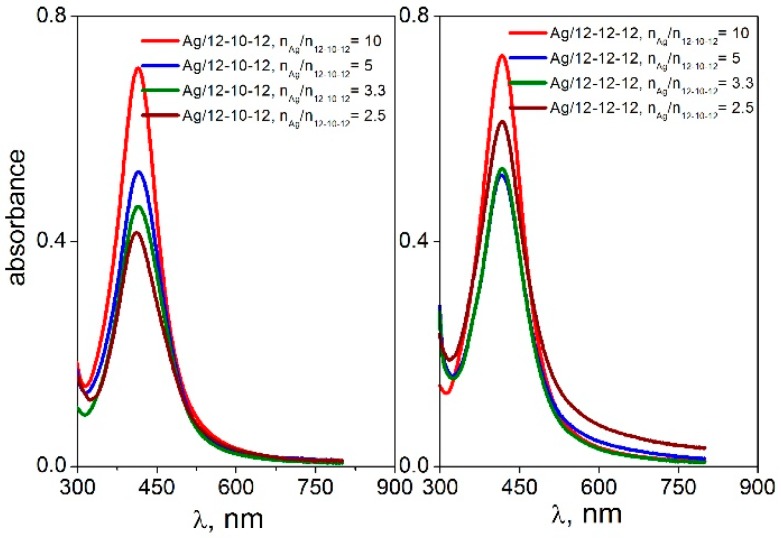
UV-VIS spectra of silver nanoparticles stabilized with gemini surfactants 12-*s*-12 with the spacer length *s* = 10, 12 methylene groups, at different molar ratio values n_Ag_/n_12-*s*-12_.

Surface plasmon oscillations of conductive electrons on the surface of a metal nanoparticle are responsible for a strong peak in the UV-VIS spectra [51] which is observed in the wavelength range 400–417 nm for silver nanoparticles. Figure 1 and Figure 2 show the UV-VIS spectra of silver nanoparticles with gemini surfactants 12-*s*-12 for all investigated spacer lengths (*s* = 2, 4, 6, 8, 10, and 12 methylene groups).

**Figure 3:**
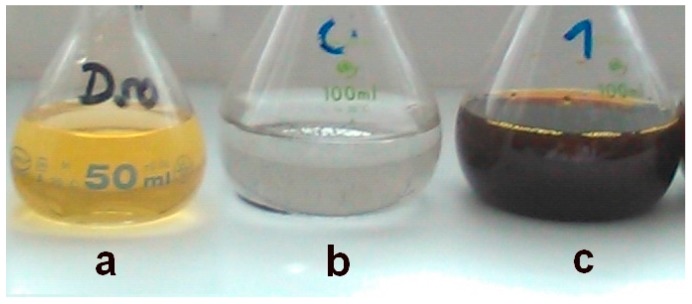
Examples of silver nanoparticle dispersions.

The spectra were recorded for four different silver-to-gemini surfactant molar ratio values. The black curve in the first plot of Figure 1 indicates the absence of gemini molecules in the dispersion. In this case, no plasmon resonance peak is found in the UV-VIS spectra. This corresponds with the observed phase separation of solid metal silver on the flask bottom with the transparent colourless layer of solvent above the solid silver (Figure 3b). As a result, the presence of gemini molecules in the silver nanodispersion provides a stabilizing effect on the nanoparticles thus keeping the nanodispersion homogeneous, with a colour ranging from dark to yellow, depending on the nanodispersion dilution (Figure 3a,c).

**Figure 4:**
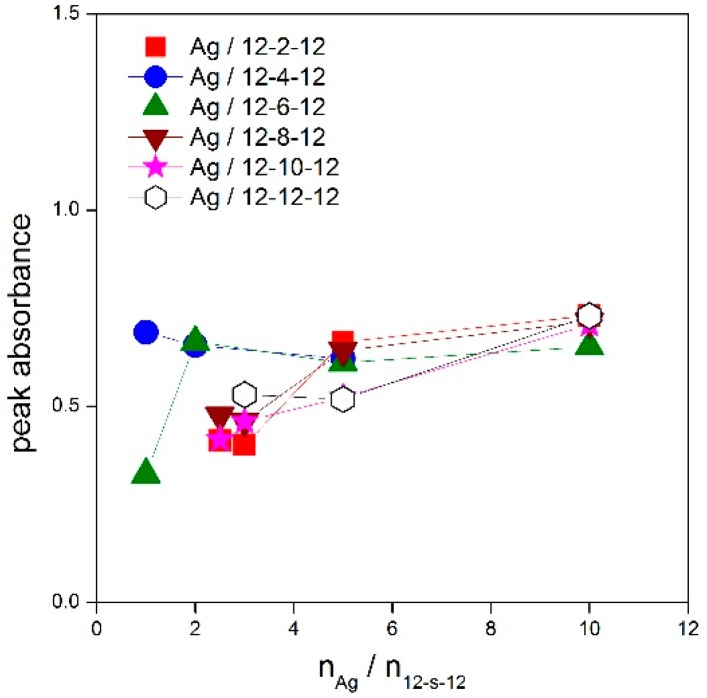
Dependence of peak absorbance on molar ratio n_Ag_/n_12-*s*-12_ for silver nanoparticles stabilized with gemini surfactants 12-*s*-12.

The peak absorbance was plotted as a function of the n_Ag_/n_12-*s*-12_ ratio for all silver nanoparticles stabilized with gemini surfactants 12-*s*-12 (Figure 4).

**Figure 5:**
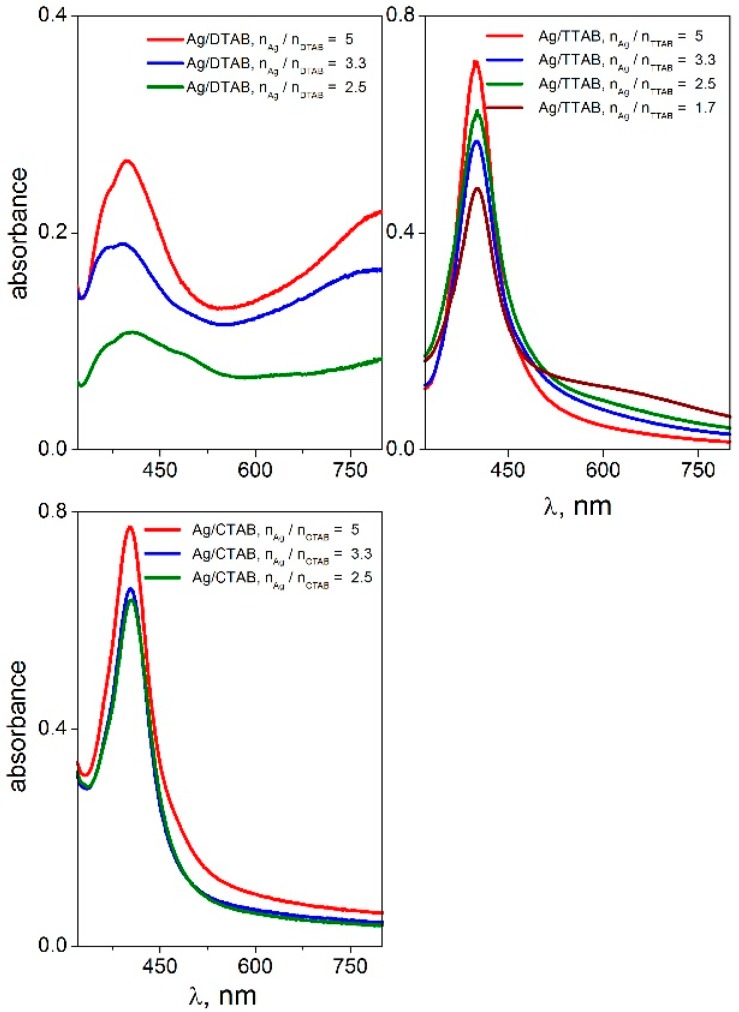
UV-VIS spectra of silver nanoparticles stabilized with single-chain surfactants DTAB, TTAB and CTAB at different molar ratio values n_Ag_/n_surf_.

The peak absorbance is independent of the silver-to surfactant molar ratio n_Ag_/n_12-*s*-12_ or moderately decreases at its low values, i.e., at higher surfactant concentration. This indicates a strong stabilizing effect of 12-*s*-12 molecules on silver nanoparticles in the wide range of n_Ag_/n_12-*s*-12_ molar ratio values with slightly less stabilizing efficiency below the ratio value of 2.5 silver atoms per gemini molecule. To document the high efficiency of gemini surfactants in stabilizing silver nanoparticles, the UV-VIS spectra of silver nanoparticles stabilized with single-chain aminium surfactants dodecyltrimethylaminium bromide (DTAB), tetratecyltrimethylaminium bromide (TTAB) and hexadecyltrimethylaminium bromide (CTAB) were recorded and plotted in Figure 5.

The highest ratio value n_Ag_/n_surf_ when the silver nanodispersion is still stable and homogeneous, was found to be only 5 (Figure 5) for single-chain surfactants as opposed to the value 10 for gemini surfactants (Figure 1 and Figure 2). In other words, the ratio of 10 silver atoms per gemini molecule results in the formation of a stabilized nanodispersion. In the case of nanoparticles stabilized with single-chain surfactants, only a maximum of five silver atoms correspond to one single-chain surfactant molecule which would form a stable nanodispersion.

The stabilizing effect of single-chain aminium surfactants decreases with the decreasing alkyl chain length as well as with the decreasing number of alkyl tails in the surfactant molecule. In Figure 5, the peak intensity of Ag/DTAB nanoparticles amounts only 0.3–0.4 while reaching values above 0.7 for Ag nanoparticles stabilized with TTAB, CTAB and gemini surfactants in Figure 1 and Figure 2. The decreasing stabilizing effect is obvious for DTAB. In the first plot of Figure 5, the peaks for Ag/DTAB nanoparticles are broad and deformed. This visually corresponded with the appearance of nanodispersions which were of light blue colour. A phase separation of solid silver was observed shortly after the preparation of Ag/DTAB nanodispersions.

### 2.2. Effect of the Gemini Surfactant Spacer Length on the Size of Silver Nanoparticles

**Figure 6:**
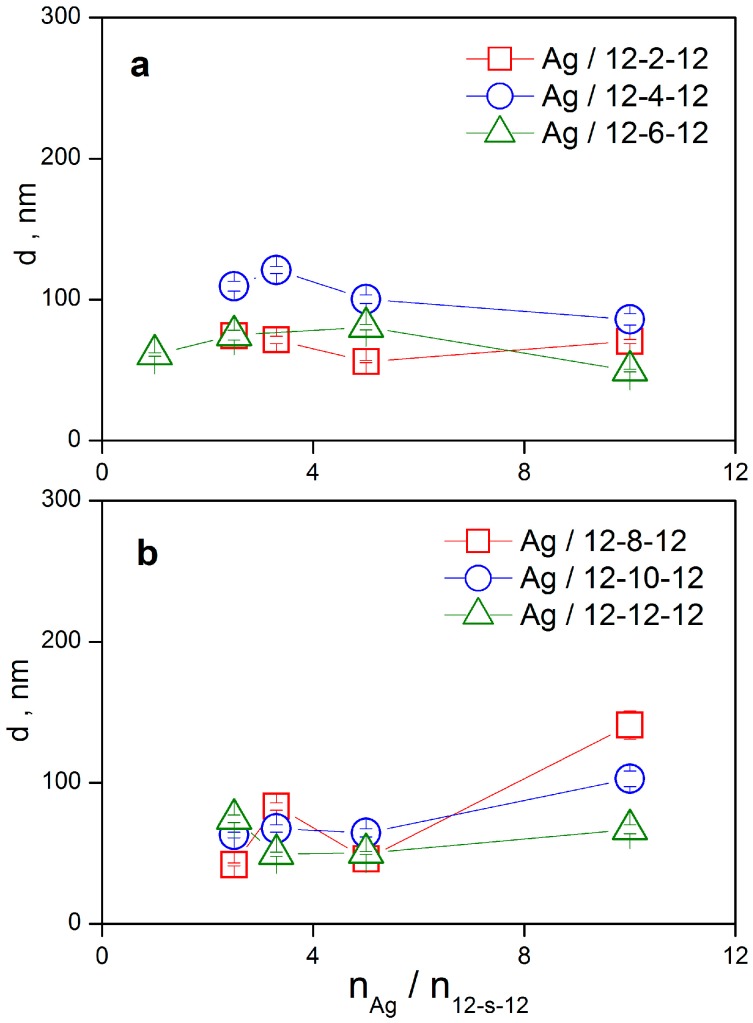
Size d of silver nanoparticles stabilized with gemini surfactants 12-*s*-12 with the spacer length *s* = 2–12 methylene groups as a function of the molar ratio values n_Ag_/n_12-*s*-12_.

The size of silver nanoparticles stabilized with gemini surfactants and their particle size distributions have been determined utilizing dynamic light scattering measurements. Hydrodynamic diameter d of silver nanoparticles stabilized with gemini surfactant molecules 12-*s*-12, *s* = 2 to 12 is plotted as a function of the molar ratio n_Ag_/n_12-*s*-12_ in Figure 6a,b. The nanoparticle diameter values were calculated from time correlation functions using the method of cumulants and represent an average value independent of the particle size distribution.

For shorter spacer values *s* = 2, 4, 6, silver nanoparticle size does not significantly depend on the silver-to-surfactant ratio and provides the values within the 50–115 nm range. A similar result indicating a constant mean nanoparticle diameter which is roughly independent of n_Ag_/n_12-*s*-12_, is observed for longer spacer values *s* = 8, 10, 12. The only exception is the largest value n_Ag_/n_12-*s*-12_ = 10 (Figure 6b), where the number of gemini molecules is too low for an efficient nanodispersion stabilization. As a result, a destabilizing effect and a nanoparticle size increase are observed. Again, the best stabilizing efficiency, even at this low gemini molecules concentration, was attained for silver nanoparticles with gemini molecules 12-12-12 having the longest spacer of 12 methylene groups. In this case, only moderate size increase of Ag/12-12-12 nanoparticles is observed at the highest ratio 10 silver atoms per gemini molecule.

**Figure 7:**
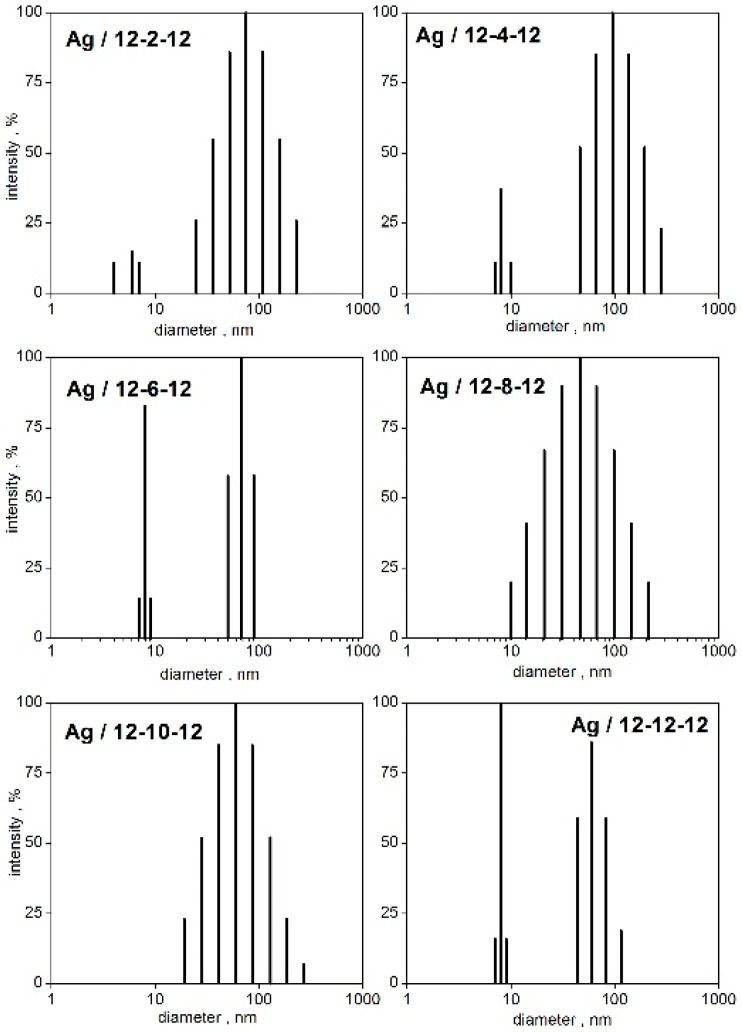
CONTIN particle size distributions of silver nanoparticles stabilized with gemini surfactants 12-*s*-12 at the molar ratio n_Ag_/n_12-*s*-12_ = 3.3.

This indicates the importance of hydrophobic interaction on stabilizing effect of cationic surfactants on silver nanoparticles, in accordance with the results from UV-VIS spectroscopy (Figure 5). To analyse the nanoparticle sizes in detail, particle size distributions from time correlation functions have been constructed using the CONTIN algorithm (Figure 7).

The time correlation functions were analysed utilizing the CONTIN algorithm at a constant molar ratio value n_Ag_/n_12-*s*-12_ = 3.3 which lies in the concentration region where stable nanodispersions were observed. As indicated in Figure 7, the size distributions have been found in most cases to be bimodal, for the shorter and long spacer lengths *s* = 2, 4, 6, and 12, respectively. In that case, two peaks are observed in the spectra. The first peak corresponds to the population of small nanoparticles with the diameter in the range 6–8 nm (Figure 7). A similar formation of small silver nanoparticles stabilized with 12-2-12 gemini surfactant with the size around 11 nm is reported in the literature [46]. It also results from the size distributions, that the population of small nanoparticles increases with the increasing spacer length in the spacer length order *s* = 2, 4, 6, 12 (Figure 7) which is documented by the increasing peak intensity. The long spacer of a gemini molecule seems to favour the population of small silver nanoparticles. For the gemini molecules with the medium spacer length *s* = 8 and 10 methylene groups, the population of small-sized nanoparticles is absent. Nanoparticle size distributions are unimodal and show a single intensity peak. The size distribution of Ag/12-8-12 nanoparticles represents a “transition state” between the unimodal and bimodal type of distributions because a broad peak with the mean particle size at around 40 nm is observed (Figure 7).

The relationship between the Ag nanoparticle size and the spacer length of a 12-*s*-12 gemini molecule seems to be related to the molecular structure and aggregation properties of 12-*s*-12 gemini surfactants in aqueous solutions. Numerous studies revealed that the aggregation characteristics such as area per surfactant molecule at the phase interface [52,53], critical micelle concentration [54], micelle aggregation number [55,56] of 12-*s*-12 gemini surfactants non-linearly depend on the spacer length.

**Figure 8:**
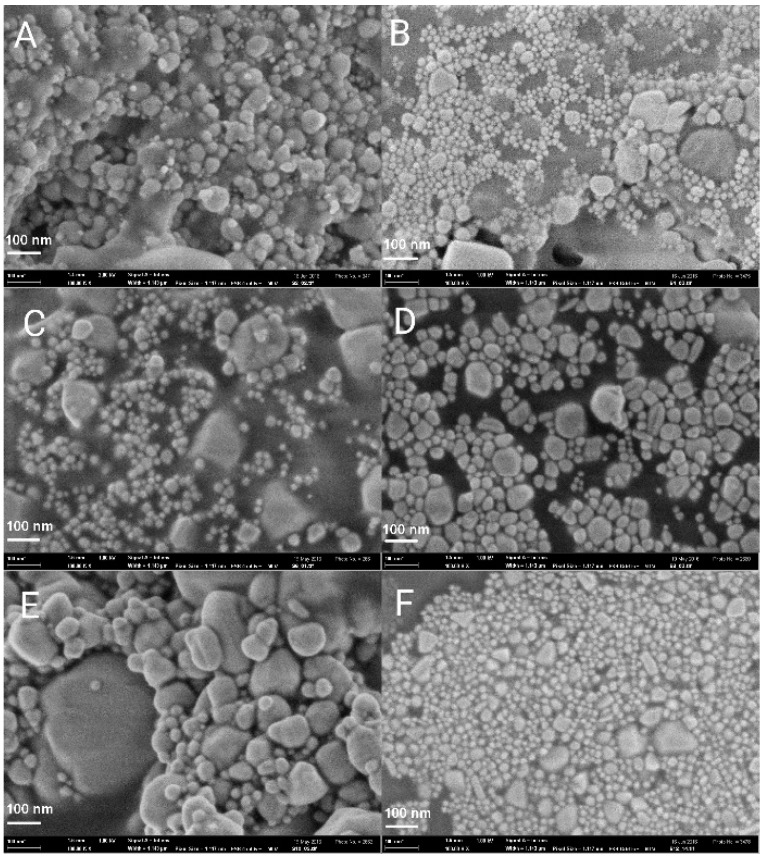
SEM images of Ag/12-*s*-12 nanoparticles. (**A**): *s* = 2, (**B**): *s* = 4, (**C**): *s* = 6, (**D**): *s* = 8, (**E**): *s* = 10, (**F**): *s* = 12.

As the results of the abovementioned studies indicate, a short spacer *s* = 2, 4 and a long spacer *s* = 12 and more, are responsible for stronger hydrophobic interactions, better adsorption at the interfaces and aggregation of gemini molecules in aqueous solution. This could be a reason for the increased stabilization effect of the gemini molecules with the spacer value 2, 4, 6, and 12 methylene groups on silver nanoparticles, as results from the nanoparticle size analysis described in the text above. The scanning electron microscopy images of silver nanoparticles stabilized with 12-*s*-12 gemini surfactants are shown in Figure 8.

As results from Figure 8, the population of the silver nanoparticles for individual spacer lengths roughly corresponds to that shown in the nanoparticle size distributions in Figure 7. The appearance of small silver nanoparticles with diameters below 10 nm is obvious for silver nanoparticles stabilized with 12-2-12, 12-4-12, 12-6-12, and 12-12-12 gemini surfactants. As for the gemini surfactant with the shortest spacer of two methylene groups, 12-2-12 (Figure 8A), the population of small nanoparticles is less intensive, which corresponds to the lower peak intensity in Figure 7 for small particles. A stabilizing effect of short-spacered gemini molecules on metal nanoparticles has been found and reported in the literature. 12-4-12 gemini molecules have been found to decrease the size of gold nanoparticles from 10 nm up to 3–4 nm with the increasing gemini surfactant concentration from 0.01 mM to 10 mM [49]. The largest population of small particles is observed for *s* = 12 which is in accordance with the particle size distribution plot in Figure 7.

### 2.3. Correlation between Gemini Surfactant Spacer Length and Zeta Potential of Silver Nanoparticles

**Figure 9:**
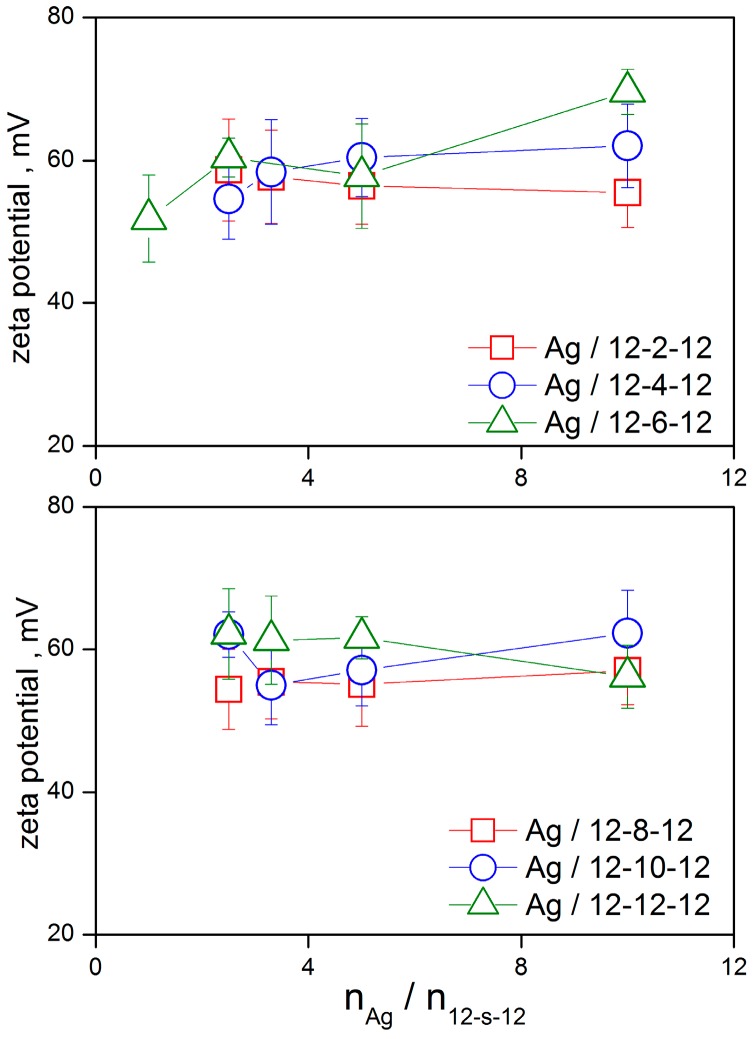
Zeta potential of silver nanoparticles stabilized with gemini surfactants 12-*s*-12 as a function of n_Ag_/n_12-*s*-12_.

The charged bilayer of gemini surfactant molecules 12-*s*-12 surrounding a silver nanoparticle core is responsible for its zeta potential value. The overall zeta potential value of a silver nanoparticle Ag/12-*s*-12 in a stable nanodispersion is positive. This is related to a significant portion of bromide counterions which are released from the outer diffuse layer of a moving Ag/12-*s*-12 nanoparticle in the electric field during the zeta potential measurement. The following plot shows the dependence of zeta potential of silver nanoparticles stabilized with gemini surfactants 12-*s*-12 on the ratio n_Ag_/n_12-*s*-12_ (Figure 9).

**Figure 10:**
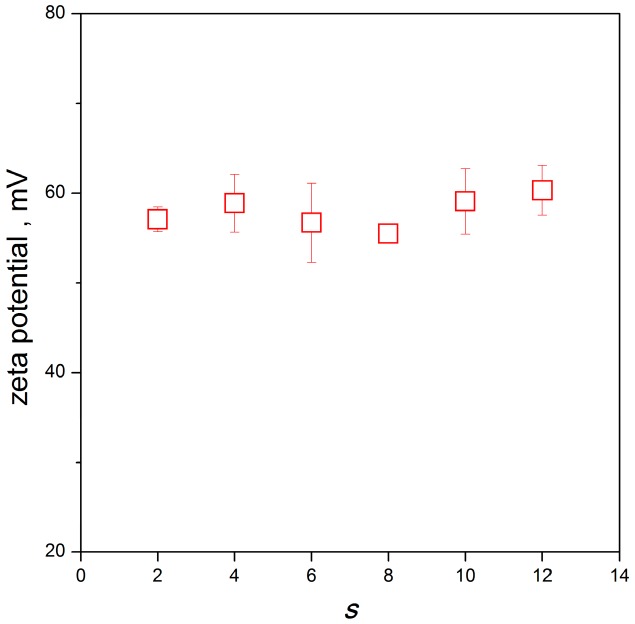
Average zeta potential of silver nanoparticles stabilized with gemini surfactants 12-*s*-12 as a function of gemini surfactant spacer length *s*.

As results from the plots, the zeta potential value of silver nanoparticles is strongly positive for all investigated spacer lengths of gemini molecules. Similarly, a positive range of zeta potential values was observed for gold nanoparticles stabilized with 12-4-12 gemini surfactant showing the zeta values between +35 and +80 mV depending on surfactant concentration [49]. Silver nanoparticles stabilized with a gemini surfactant composed of hexadecyl chains and a 3-oxapentane-1,5-diyl spacer provided the zeta potential value of +31.4 mV [47]. The plots in Figure 9 indicate that zeta potential of Ag/12-*s*-12 nanoparticles depends little on the silver-to-surfactant ratio. Like the Ag/12-*s*-12 nanoparticle size dependence, the stability of nanoparticles is slightly worse at the high n_Ag_/n_12-*s*-12_ value, i.e., at the low amount of gemini surfactant in a silver nanoparticle when the zeta potential values are more scattered. However, zeta values are sufficiently positive for all gemini spacer lengths and concentrations which indicates a strong stabilizing effect of all investigated 12-*s*-12 gemini molecules on silver nanoparticles. Following these findings, it is possible to average the concentration-dependent values of zeta potential and plot them as a function of gemini spacer length *s* (Figure 10).

As results from the plot in Figure 10, the zeta potential average value for silver nanoparticles is roughly independent of gemini surfactant spacer length and is approx. +58 mV. A slightly higher average values are observed for the short and long spacer lengths *s* = 4 and *s* = 12, respectively, which corresponds to the better stabilization efficiency of these geminis manifested by the formation of small-sized silver nanoparticles.

### 2.4. Interaction Model between 12-s-12 Gemini Molecules and Silver Nanoparticles

**Figure 11:**
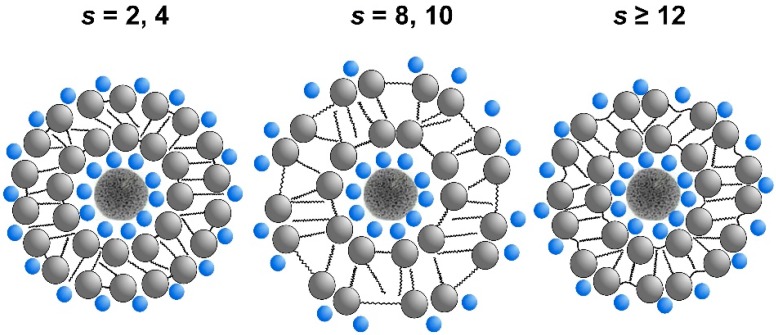
Interaction model between gemini molecules 12-*s*-12 with variable spacer length *s* and a silver nanoparticle. Bromide counterions are represented by smaller blue spheres.

Following the size analysis and zeta potential results of Ag/12-*s*-12 nanoparticles, an interaction model between gemini surfactant molecules and a silver nanoparticle is shown in the following scheme (Figure 11) which reflects the influence of the spacer length of a gemini surfactant molecule of the nanoparticle size.

A silver nanoparticle is surrounded by a bilayer composed of gemini molecules 12-*s*-12. As the model indicates, the structure of the bilayer and hence, the size of the Ag/12-*s*-12 nanoparticle depend on the gemini surfactant spacer length. A short spacer of two to four CH_2_ groups (Figure 11, *s* = 2, 4) results in a dense arrangement of gemini molecules at the air/water interface or in a gemini micelle. According to [55], there are two characteristic distances in a micelle of gemini molecules with polymethylene spacer. A structural distance d_s_ which corresponds to the extended length of the spacer. Its value for a short spacer (e.g., *s* = 2) is d_s_ = 0.38 nm, according to Tanford’s formula d_s_ = 0.1265(*s* + 1) [57].

The second distance is a thermodynamic equilibrium distance d_T_ characterizing the displacement of surfactant molecules at the interface or in a micelle which is about 0.7–0.9 nm [55]. If the spacer is short enough, it is fully stretched and the displacement distance is identical with the structural distance d_s_. The displacement of gemini molecules in the bilayer is entirely controlled by the length of their short spacer. As a result, area per surfactant is small and gemini molecules are densely packed in the nanoparticle bilayer which allows a high number of gemini molecules to be bound to the silver nanoparticle surface. These results in the formation of silver nanoparticles of a small size stabilized by a bilayer which is composed of densely arranged gemini molecules (Figure 11, *s* = 2, 4).

For a medium size spacer length *s* = 8–10, the spacer is longer, but still rigid and unable to bend into the hydrophobic phase such as micelle core or nanoparticle bilayer interior. This results in an increase of the area per surfactant at the nanoparticle surface and the decrease of gemini molecules density in the nanoparticle bilayer. At this spacer length, the structural distance d_s_ approaches the thermodynamic distance d_T_ of the displacement of gemini molecules [55]. The formed silver nanoparticles show size increase and the stabilization effect of gemini bilayer is less efficient due to the smaller number of gemini molecules forming the bilayer (Figure 11, *s* = 8, 10). In the case of a long spacer *s* = 12 and more, the spacer is considered to be flexible and able to be bent into the surfactant bilayer interior [52]. This results in a decrease of the distance separating aminium polar groups in a gemini molecule and the subsequent decrease of the area per surfactant value. Similarly to the case of short spacer, the density of gemini molecules in the bilayer increases. The silver nanoparticles show smaller size and higher number of bound gemini molecules to the nanoparticle surface (Figure 11, *s* ≥ 12).

### 2.5. Microbicidal Activity of Silver Nanoparticles Stabilized with Gemini Surfactants

**Figure 12:**
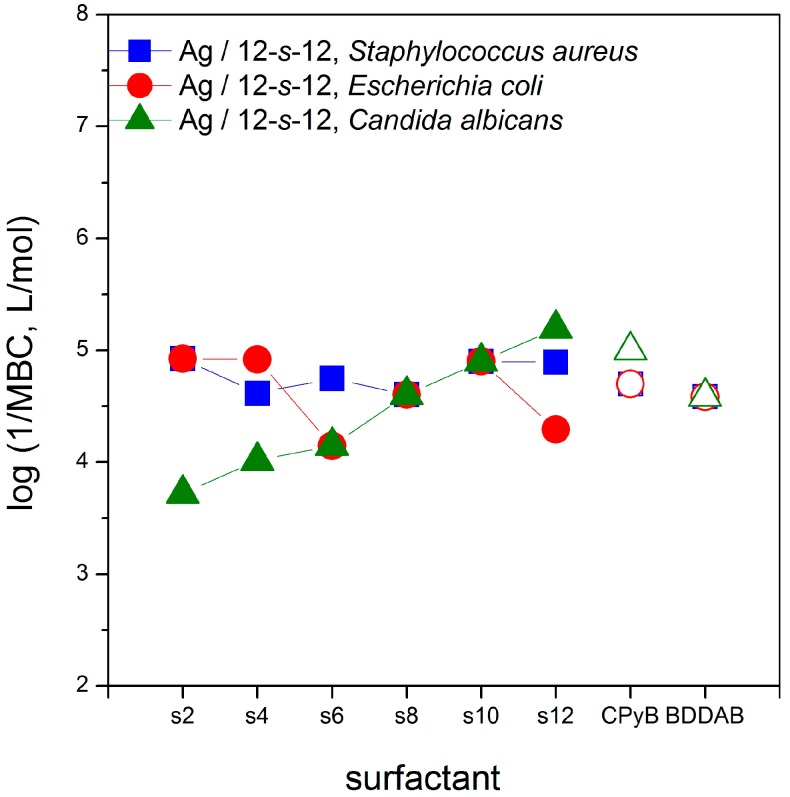
Logarithm of 1/MBC plotted against the gemini spacer length *s* for gram-positive bacteria, gram-negative bacteria and yeast. Reference samples are plotted with the open symbols.

The microbicidal activity of silver nanoparticles stabilized with gemini surfactants 12-*s*-12 was evaluated against Gram-positive bacteria, Gram-negative bacteria and yeast by determining the minimum bactericidal concentration (MBC) utilizing the diffusion agar technique. The microbicidal efficiency is expressed as the logarithm of inverse MBC values and is plotted in Figure 12 as a function of the gemini surfactant spacer length. The last two points in the plot indicate the microbicidal activity of the usual reference compounds cetylpyridinium bromide (CPyB) benzyldodecyldimethylaminium bromide (BDDAB).

The comparison of log 1/MBC values of Ag/12-*s*-12 nanoparticles plotted in Figure 12 with those for Ag nanoparticles without gemini molecules [58] provides better microbicidal activity values for Ag/12-*s*-12 nanodispersions. The microbicidal activity values for pure Ag nanoparticles are 464 μM (log(1/MBC) = 3.3) and 116 μM (log(1/MBC) = 3.9) for bacteria *S. aureus* and *E. coli*, respectively [58]. As shown in Figure 12, the microbicidal activity does not significantly depend on the spacer length for Gram-positive and Gram-negative bacteria. This in accordance with the experimental findings that microbicidal efficiency is primarily controlled by the variation of the length of alkyl chains and, to a lesser extent, by the length of the spacer. As the reported data indicate, the dependence of microbicidal activity on the alkyl chain length is strongly non-linear and provides an optimum length of the alkyl chain of a gemini molecule at which the maximum biological activity is attained [59,60,61].

**Figure 13:**
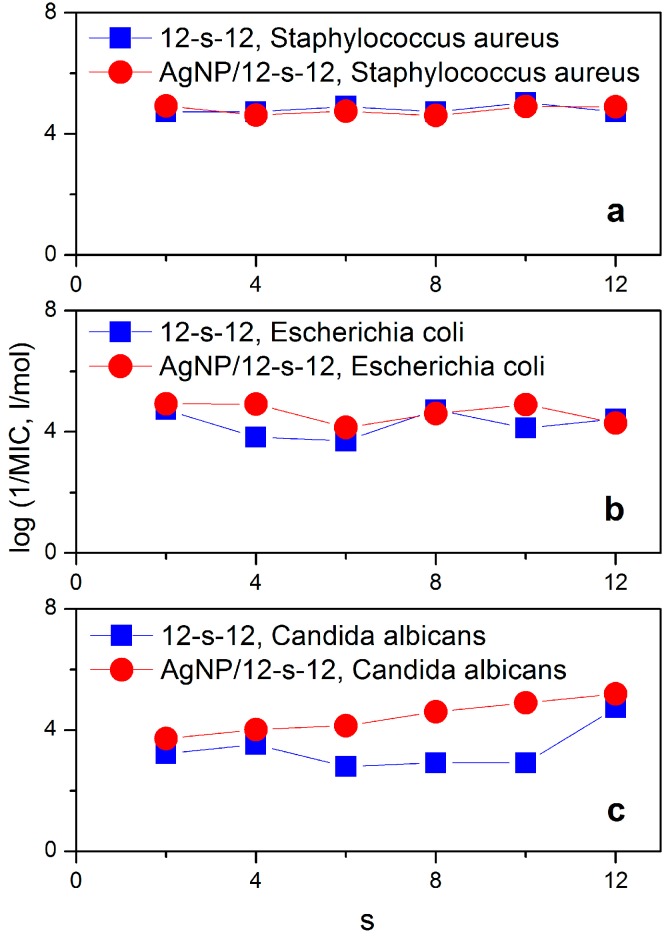
Comparison of microbicidal activity of Ag/12-*s*-12 nanoparticles and gemini surfactants 12-*s*-12 against individual bacterial strains and yeast.

A dependence of microbicidal activity on the gemini spacer length has been found for the yeast *C. albicans*. The dependence increases monotonically and the most efficient gemini surfactant is that with the longest spacer 12-12-12. To analyse the impact of the combination of silver nanoparticles and gemini surfactant molecules on microbicidal efficiency more thoroughly, microbicidal activity of gemini surfactants 12-*s*-12 themselves (without the presence of silver) was also investigated. Considering a strong microbicidal effect of both silver and cationic surfactants in general, additional information on the microbicidal activity of gemini surfactants allows to analyse a possible synergic effect in the microbicidal activity of Ag/12-*s*-12 nanoparticles. Figure 13 shows the differences in log 1/MBC vs. spacer length dependence between Ag/12-*s*-12 nanoparticles and 12-*s*-12 gemini surfactants themselves.

No significant difference is observed between the microbicidal activity of gemini surfactants 12-*s*-12 and nanoparticles Ag/12-*s*-12 against the Gram-positive bacterium *S. aureus* (Figure 13a). However, nanoparticles Ag/12-*s*-12 have been found to be more microbicidally efficient against the Gram-negative *E. coli* and the yeast *C. albicans* than the gemini surfactants themselves. The possible explanation of the decreased susceptibility of Gram-positive bacteria to Ag^+^ ions as compared with Gram-negative bacteria, is related to their cell wall structure and thickness. The peptidoglycan-multilayered cell wall of Gram-positive bacteria is thicker than the single-layered cell wall of Gram-negative bacteria which hampers the penetration of small silver cations. The multiple layers of Gram-positive bacteria contain substantially more peptidoglycan molecules (3- to 20-fold higher) than the cell walls of Gram-negative bacteria [16]. Due to the fact that peptidoglycans are negatively charged, they may bind some portion of the silver. Accordingly, Gram-positive bacteria may allow less silver ions to reach the cell membrane to perform their microbicidal effect [62]. In this way, the microbicidal effect against gram-positive bacteria which is shown in the upper plot in Figure 13, arises only from gemini surfactants 12-*s*-12 with no significant contribution of silver nanoparticles.

In the case of less amount of peptidoglycans in a cell wall (*E. coli*) and or their absence (i.e., in the cell wall of *C. albicans*), the microbicidal effect of the presence of silver is noticeable. The curves for the microbicidal activity of silver nanoparticles stabilized with gemini surfactants Ag/12-*s*-12 are above those for gemini surfactants (Figure 13b,c), thus indicating a better microbicidal effect of silver nanoparticles with gemini surfactants.

## 3. Experimental Section

### 3.1. Chemicals

**Figure 14:**
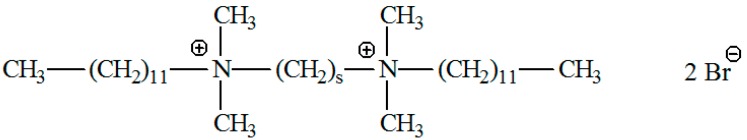
Molecular structures of the gemini surfactants 12-*s*-12.

Cationic bis-aminium gemini surfactants with the polymethylene spacer alkanediyl-α,ω-bis(dimethyldodecylaminium bromides), hereinafter referred to as 12-*s*-12 (*s* is the number of CH_2_ groups in the spacer, Figure 14), were prepared as described in the literature [63].

For the synthesis of gemini surfactants with the spacer number *s* = 2, 6, 8, and 10, a reaction of *N*,*N*,*N′*,*N′*-tetramethylalkane-α,ω-diamine with 1-bromododecane was applied. A reaction of *N,N*-dimethyldodecane-1-amine with -α,ω-dibromoalkane was used for the synthesis of gemini surfactants with the spacer number *s* = 4 and 12. The products were purified by manifold crystallization from an acetone-methanol mixture [63].

### 3.2. Preparation of Silver Nanoparticles Stabilized with Gemini Surfactants

Specific amounts of gemini surfactants 12-*s*-12 corresponding to four different molar ratio values n_Ag_/n_12-*s*-12_ = 10, 5, 3.3, 2.5, and of silver nitrate (concentration 5 mM) were weighed and dissolved in 40 mL deionized water and stirred for 5 min until a silver bromide sol was formed. Separately, a 100 mL sodium borohydride stock solution was prepared at the NaBH_4_ concentration 15 mM. Using an automated burette (765 Dosimat, Metrohm, Herisau, Switzerland), NaBH_4_ stock solution (10 mL) was added to the AgNO_3_/12-*s*-12 stirred solution, at a slow, controlled flow rate of 1 drop per second to assure the formation of stable silver nanodispersions with narrow nanoparticle size distribution. After adding the whole volume of sodium borohydride solution, the colour of the solutions changed from yellow to dark, thus indicating the formation of gemini surfactant-stabilized nanodispersions. Prior to the measurements, the silver nanodispersions were left for three days to age. As a control, unmodified silver nanodispersion was prepared by same procedure in aqueous solution without a gemini surfactant stabilizer. However, a phase separation occurred immediately after the preparation.

### 3.3. UV-VIS Spectroscopic Characterization of Silver Nanoparticles

UV-VIS absorption spectra were recorded at 25 °C on a Genesys 10S UV–VIS spectrophotometer (ThermoFischer Scientific, Waltham, MA, USA) operated by the VISIONLite spectrophotometer software (ThermoFischer Scientific, Waltham, MA, USA) using quartz cuvettes of 1 cm path length. To decrease the absorbance of the prepared silver nanodispersions to the range of values which are suitable for the UV-VIS spectrophotometric measurements, the samples were diluted 50-fold with distilled water.

### 3.4. Dynamic Light Scattering

The hydrodynamic diameter of silver nanoparticles was determined using a dynamic light scattering equipment (Brookhaven BI 9000 digital correlator, Brookhaven SM 200 goniometer, Lexel argon laser, Brookhaven Instruments Corporation, Holtsville, NY, USA). The argon laser at 514.5 nm wavelength was used as the incident light source. The intensity time fluctuations of the scattered light were detected at a scattering angle of 90° and a temperature 25 °C and autocorrelated in the BI 9000 correlator card where the time correlation function was built. The translation diffusion coefficient was calculated from the time correlation function using the method of cumulants. The method of cumulants in dynamic light scattering data analysis was introduced by Koppel [64] who showed that the decay rate $\bar{\Gamma }$ is proportional to the z-average of the diffusion coefficient D as follows:

(1) $$\bar{\Gamma }=q^{2}D$$

The logarithm of the electric field autocorrelation function g^(1)^(t) can be expanded into a series as follows:

(2) $$\ln(g^{\left(1\right)}(t))=-\bar{\Gamma }t+\frac{1}{2!}\mu_{2}t^{2}\;–\;.....$$

μ_2_ is the second cumulant which would be near zero for a perfect single exponential decay. The decay term $\bar{\Gamma }$ was calculated from the expansion of the logarithm of time correlation function up to the second term using the first linear term in Equation (2) for the calculation. The diffusion coefficient was determined using Equation (1) from the decay rate and the hydrodynamic diameter d was calculated from the diffusion coefficient using Stokes–Einstein formula:

d = kT/(3πηD)(3)

where η is the solvent viscosity, k is the Boltzmann constant, and T is the absolute temperature. Five independent measurements and calculations of time correlation function were carried out for each gemini surfactant spacer length and the silver-to-surfactant molar ratio was investigated. The mean value and standard deviation of d for each sample were calculated.

To determine nanoparticle size distributions, a numerical algorithm based on the inverse Laplace transformation was applied on the time correlation function. Nanoparticle diameters were evaluated from the partition size distributions utilizing the CONTIN algorithm [65] with the rejection probability parameter set to 0.5. Due to the large number of processed data, a custom application software written in Visual Basic was used for automated data format conversion from the measurement files.

### 3.5. Zeta Potential Measurements

Zeta potential measurements were performed with the Brookhaven BI ZetaPlus equipment which is based on the measurement of the electrophoretic mobility utilizing the Doppler frequency shift. Zeta potential values were calculated from the measured mobility using the Smoluchowski limit for the mobility vs. zeta potential relationship. The mean value was calculated from a statistical set of 20 zeta potential recordings per each molar ratio value n_Ag_/n_12-*s*-12_ and each spacer length. The measurements were taken at 25 °C.

### 3.6. Scanning Electron Microscopy

The samples of gemini surfactants were analyzed using a scanning electron microscope equipped with an EDX analyser (Zeiss EVO LS10, Zeiss, Oberkohen, Germany) using accelerating voltage 10.0 kV, working distance 8.0 mm and probe current 80 pA. Au sputtering was applied to avoid unfavourable sample conductivity.

### 3.7. Determination of Microbicidal Activity

The microbicidal activity of Ag/12-*s*-12 nanoparticles was evaluated in vitro as the minimum bactericidal concentration (MBC). It was determined using the standard broth dilution method [66]. The following representative Gram-positive and Gram-negative bacteria and yeast were selected for the experiments: *Staphylococcus aureus ATCC 6538*, *Escherichia coli ATCC 11229* and *Candida albicans CCM 8186*. Two commercially used quaternary aminium disinfectants, cetylpyridinium bromide (CPB) and benzyldodecyldimethylaminium bromide (BDDAB), were used as the reference compounds. The determination of their MBC values as well as the method of testing are described in our previous papers [66,67].

## 4. Conclusions

The present study provides information of the preparation and the investigation of physicochemical and biological properties of silver nanoparticles that are stabilized with a series of cationic gemini surfactants with polymethylene spacers of variable length. Physicochemical investigations utilized several methods such as UV-VIS spectroscopy, nanoparticle size determination using dynamic light scattering and scanning electron microscopy methods and the measurements of zeta potential of nanoparticles. Biological properties are represented by the determination of the microbicidal activity of the silver nanoparticles with gemini surfactants. For the sake of comparison, the microbicidal activity of aqueous solutions was determined as well. It follows from the experimental data, that the investigated gemini molecules at all spacer length values are able to efficiently stabilize silver nanoparticles. However, the best stabilization, which is reflected by a small nanoparticle size, was attained by the application of gemini surfactants with either a short methylene spacer (2, 4 –CH_2_– groups) or long spacer (12 –CH_2_– groups). The proposed interaction model between silver nanoparticles and gemini molecules reflects the collected experimental data. Microbicidal activity determination revealed that silver nanoparticles stabilized with gemini surfactants are more efficient against Gram-negative bacteria and yeast, which has a direct relation to the mechanism of the interaction of nanoparticles with the microbial cell membrane and its structural composition. The presented study constitutes a solid basis for further investigations of the relationship between surfactant molecular structure and the composition, size and stability of silver nanoparticles.
